# The canonical TGF-β/Smad signalling pathway is involved in PD-L1-induced primary resistance to EGFR-TKIs in EGFR-mutant non-small-cell lung cancer

**DOI:** 10.1186/s12931-019-1137-4

**Published:** 2019-07-22

**Authors:** Yang Zhang, Yuanyuan Zeng, Ting Liu, Wenwen Du, Jianjie Zhu, Zeyi Liu, Jian-an Huang

**Affiliations:** 1 Department of Respiratory Medicine, the First Affiliated Hospital of Soochow University, Suzhou, 215006 People’s Republic of China; 2 Suzhou Key Laboratory for Respiratory Diseases, Suzhou, 215006 China; 3 Institute of Respiratory Diseases, Soochow University, Suzhou, 215006 China

**Keywords:** PD-L1, EGFR-TKI, Drug resistance, TGF-β/Smad signalling, NSCLC

## Abstract

**Background:**

Approximately 30% of patients with epidermal growth factor receptor (EGFR)-activating mutations have no response to EGFR-tyrosine kinase inhibitors (TKIs) (primary resistance). However, little is known about the molecular mechanism involved in primary resistance to EGFR-TKIs in EGFR-mutant non-small cell lung cancer (NSCLC). Programmed death ligand-1 (PD-L1) plays important regulatory roles in intracellular functions and leads to acquired resistance to EGFR-TKIs in NSCLC. Here, we investigated the mechanistic role of PD-L1 in primary resistance to EGFR-TKIs in EGFR-mutant NSCLC cells.

**Methods:**

The expression levels of PD-L1 and the sensitivity to gefitinib in H1975, HCC827 and PC-9 cells were determined by quantitative real-time PCR analysis (qRT-PCR) and Cell Counting Kit-8 (CCK-8) assays, respectively. Molecular manipulations (silencing or overexpression) were performed to assess the effect of PD-L1 on sensitivity to gefitinib, and a mouse xenograft model was used for in vivo confirmation. Western blotting and qRT-PCR were used to analyse the expression of epithelial-mesenchymal transition (EMT) markers. The effect of PD-L1 on migratory and invasive abilities was evaluated using the Transwell assay and mice tail intravenous injection.

**Results:**

Higher expression of PD-L1 was related to less sensitivity to gefitinib in EGFR-mutant NSCLC cell lines. The overexpression or knockdown of PD-L1 presented diametrical sensitivity to gefitinib in vitro and in vivo. Furthermore, the overexpression of PD-L1 led to primary resistance to gefitinib through the induction of EMT, which was dependent on the upregulation of Smad3 phosphorylation. Moreover, in the mouse model, the knockdown of PD-L1 inhibited transforming growth factor (TGF)-β1-induced cell metastasis in vivo.

**Conclusion:**

PD-L1 contributes to primary resistance to EGFR-TKI in EGFR-mutant NSCLC cells, which may be mediated through the induction of EMT via the activation of the TGF-β/Smad canonical signalling pathway.

## Introduction

Lung cancer has long been the leading cause of cancer-related death worldwide [1]. Approximately 80% of all lung cancer cases are non-small cell lung cancer (NSCLC) [2]. Epidermal growth factor receptor (EGFR) is a key tumour driver, and the EGFR signalling pathway has been shown to be a main target in the successful treatment of NSCLC [3–6]. Among patients with EGFR-activating mutations, approximately 70% exhibit objective responses to EGFR-tyrosine kinase inhibitors (TKIs) [7, 8]. Nevertheless, approximately 30% of patients with EGFR-activating mutations do not respond to EGFR-TKIs (primary resistance) [5, 6]. Currently, the mechanism of primary resistance is not fully understood beyond genomic mechanisms, including the coexisting de novo T790 M mutation [9], de novo mesenchymal-epithelial transition (MET) amplification [10], phosphatase and tensin homologue (PTEN) loss [11] and Kirsten rat sarcoma viral oncogene homologue (KRAS) mutations [12]. Therefore, further studies are required to clarify the mechanisms of primary resistance.

PD-L1 (B7-H1, CD274) is an important immune co-signalling molecule from the B7/CD28 family [13]. PD-L1 negatively regulates T cell functions through interactions with PD-1 and CD80 [14]. Numerous works have shown that PD-L1 regulates the biological behaviours of cancer cells independently of cytotoxic T cells and PD-1. For instance, PD-L1 regulates tumour glucose metabolism [15], reduces chemotherapy-mediated tumour killing by modifying mitogen-activated protein kinase signals [16], and prevents cell proliferation and apoptosis [17]. Several previous studies have revealed the relationship between EGFR signalling pathways and PD-L1. The presence of activated EGFR signalling increased the expression of PD-L1 [18–20]. Surgically resected specimens from advanced NSCLC patients with EGFR mutations demonstrated that EGFR mutation is associated with high PD-L1 expression [21]. Furthermore, higher PD-L1 expression has been detected in patients with acquired resistance to EGFR-TKIs [22]. Although the possible mechanisms by which PD-L1 leads to acquired resistance to EGFR-TKIs in NSCLC, including the upregulated expression of YAP1 and BAG-1 [23, 24], have been investigated in several studies, little is known about the relationship between PD-L1 and primary resistance to EGFR-TKIs or the potential molecular mechanism.

The epithelial-to-mesenchymal transition (EMT) decreases the clinical activity of gefitinib and erlotinib and the sensitivity of NSCLC cells to these drugs [25, 26], and the transforming growth factor (TGF)-β/Smad signalling pathway plays an important role in EMT progression in various epithelial cell types [27, 28]. Smad3 is a key regulator of the canonical TGF-β signalling pathway and an important checkpoint in TGF-β1-mediated transcriptional regulation [29, 30]. One recent study indicated that PD-L1 promoted malignant transformation and mediated the regulation of EMT in human oesophageal cancer [31]. Therefore, we hypothesized that PD-L1 confers primary resistance to EGFR-TKIs in EGFR-mutant NSCLC via the upregulation of Smad3 phosphorylation.

In this study, we aimed to investigate the relationship between PD-L1 and primary resistance to EGFR-TKIs in EGFR-mutant NSCLC cells. Furthermore, we revealed the mechanism by which PD-L1 generates primary resistance to EGFR-TKIs and evaluated the role of Smad3 in primary resistance to EGFR-TKIs induced by PD-L1 expression.

## Materials and methods

### Cell culture and reagents

The EGFR-mutant human lung adenocarcinoma cell lines HCC827 and H1975 were purchased from the Cell Bank of the Chinese Academy of Sciences (Shanghai, China). The cells were seeded and grown in RPMI 1640 medium (Gibco) with 10% FBS (Gibco) and 1% penicillin/streptomycin (Gibco). PC-9 cells were obtained from Professor Cai-cun Zhou as a gift and were seeded in DMEM (HyClone, South Logan, UT, USA) supplemented with 10% FBS (Gibco) and 1% penicillin/streptomycin (Gibco). All cells were cultured in humidified incubators at 37 °C with 5% CO_2_.

Gefitinib and SIS3 were purchased from Selleck Chemicals (Houston, TX, USA). Human TGF-β1 was purchased from R&D Systems (Minneapolis, MN, USA). Mouse TGF-β1 was purchased from Novoprotein Scientific (Summit, NJ, USA).

### Cell viability assay

Tumour cells (3 × 10^3^) were plated in each well of a 96-well plate, grown overnight and then treated with varying drug concentrations for 72 h: 0, 0.001, 0.01, 0.05, 0.1, 0.5, 1, 5, 10 and 20 μM gefitinib. Each well was treated with 10 μl of Cell Counting Kit-8 (CCK-8) solution (Beyotime, SongJiang, Shanghai, China), and the OD450 was measured by a microplate reader after 4 h (Thermo, Waltham, MA, USA).

### Establishment of stable PD-L1-overexpressing cell lines

To generate HCC827 cells in which PD-L1 is stably overexpressed, we subcloned the coding sequence of PD-L1 into a pLVX-IRES-Neo vector using the endonucleases EcoRI and XbaI for expression by a Lenti-X lentiviral expression system (Clontech, Mountain View, CA, USA). The empty vector was used as a negative control. The PD-L1 expression construct was co-transfected with packaging plasmids into human embryonic kidney 293 T cells using Lipofectamine 2000 (Invitrogen, Waltham, MA, USA). Human embryonic kidney 293 T cells were cultured in DMEM with 10% foetal bovine serum at 37 °C in a humidified 5% CO_2_ incubator for 48 h. After incubation, the packaged lentiviruses were collected and used to infect HCC827 cells. After 2 days, stable cells were selected with 400 μg/ml G418 (Amresco, Solon, OH, USA). The coding sequence for PD-L1 was amplified using the following primers: forward, 5′- GGTGCCGACTACAAGCGAAT-3′; reverse: 5′- GGTGACTGGATCCACAACCAA − 3′.

### Establishment of stable PD-L1-silenced cell lines

To establish stable cell lines with silenced PD-L1 expression, two DNA fragments (PD-L1 ShRNA-1, 5′-GCATTTGCTGAACGCATTTAC-3′; PD-L1 ShRNA-2, 5′-CGAATTACTGTGAAAGTCAAT-3′) were subcloned into the lentiviral vector pGLV2-U6-Puro (GenePharma, Shanghai, China) containing the endonucleases BamHI and EcoRI. A scrambled PD-L1 ShRNA sequence served as the negative control. Then, the PD-L1-silenced construct or the negative control was co-transfected with packaging plasmids into human embryonic kidney 293 T cells using Lipofectamine 2000 (Invitrogen). After 48 h, the cells were infected with the packaged lentiviruses and cultured for 2 days before being selection with 0.4 μg/ml or 2 μg/ml puromycin (Sigma-Aldrich, St. Louis, MO, USA).

### Western blotting

The cells were grown to 80~90% confluence and then lysed in RIPA buffer (Cell Signaling Technology, Danvers, MA, USA) with protease inhibitor and phosphatase inhibitor cocktail (Sigma-Aldrich, St. Louis, MO, USA).The cell lysates were cleared by centrifugation at 4 °C for 20 min at 12000 x g. Total cell lysates were separated by 10% SDS-PAGE, transferred to nitrocellulose membranes (Millipore, Billerica, MA, USA), blocked with 5% milk in TBS buffer with 0.1% Tween-20 (TBST) for 1 h at room temperature and incubated with primary antibodies overnight at 4 °C. After washing four times with TBST, the membranes were incubated with the corresponding HRP-conjugated secondary antibodies for 2 h at room temperature. Detection was performed using an ECL kit (Thermo Fisher Scientific, Waltham, MA, USA). The band density was quantified using Quantity One 4.6 software. All antibodies used for western blotting, including anti-Smad3, anti-PD-L1, anti-β-actin antibodies and anti-mouse and anti-goat secondary antibodies were obtained from Cell Signaling Technology.

### RNA extraction and quantitative real-time PCR analysis

Total RNA was extracted from cells by adding 1.0 ml of RNAiso Plus (Takara, Osaka, Japan), according to the manufacturer′s protocol. The RNA concentration was measured using a NanoDrop 2000 (Thermo Fisher Scientific, Waltham, MA, USA). The synthesis of cDNA was carried out with reverse transcriptase M-MLV (Takara). The primer sequences for the qRT-PCR of PD-L1 and β-actin (ACTB) were as follows: PD-L1, forward:5-'GGTGCCGACTACAAGCGAAT-3′, reverse: 5′-GGTGACTGGATCCAC AACCAA-3′; ACTB, forward:5′-CACAGAGCCTCGCCTTTGCC-3′, reverse:5′-ACCCATGCCCACC ATCACG-3′. qRT-PCR was performed using SYBR Premix ExTaq™ (Takara), according to the manufacturer’s instructions, with an ABI Step One Plus Real-Time PCR system (Applied Biosystems, Foster City, CA, USA). The PCR program was 50 °C for 2 min and 95 °C for 10 min, followed by 45 cycles of 95 °C for 15 s and 60 °C for 1 min. The mRNA expression of PD-L1 were normalized to the internal control ACTB. Relative expression was calculated using the cycle threshold (Ct) method.

### Xenografts

BALB/c athymic nude mice (female, 4–6 weeks old and 16–20 g) were purchased from the Experimental Animal Center of Soochow University and bred under pathogen-free conditions. Three million stable PD-L1 cells (HCC827 PLVX/PD-L1) were subcutaneously injected into the nude mice. When the tumour volumes reached approximately 70–100 mm^3^, the mice were randomly allocated into groups of four animals and administered gefitinib 50 mg/kg or vehicle by oral gavage every 2 days for 16 days. Tumour growth was analysed by measuring tumour length (L) and width (W) and calculating the volume (V) was calculated with the formula V = LW^2^/2. All animal experiments were carried out in accordance with the Guide for the Care and Use of Experimental Animals from the Experimental Animal Center of Soochow University.

### Migration and invasion assays

For the migration assay, 8 × 10^4^ tumour cells in medium containing 1% FBS were seeded onto the upper chamber of a Transwell insert, and 800 μl of medium containing 10% FBS was added to the lower chamber. For the invasion assay, 10× 10^4^ tumour cells in medium containing 1% FBS were seeded onto the upper chamber of a Transwell insert coated with Matrigel matrix (BD Science, Sparks, MD, USA), and 800 μl of medium containing 20% FBS was added to the lower chamber. At 6 h later, TGF-β1 or SIS3 was added to the lower chambers, and the plates were incubated at 37 °C for 24 h, according to the manufacturer’s instructions. In both assays, the cells were photographed and counted.

### Luciferase reporter assays

The − 960 to + 124 promoter region of PAI-1 was amplified by PCR (primers: forward, 5′-CGGGGTACCGCACACCCTGCAAACCTGCC-3′ and reverse, 5′-CCGCTCGAGCGATTGGCGGT TCGTCCTG-3′). The products were then digested with KpnI and XhoI, and the obtained fragments were directly ligated into the pGL3 basic vector (Promega). The empty pGL3 vector (800 ng), pGL3-PAI-1(800 ng) and pRL-TK (32 ng) were co-transfected with the control (Si-NC) or PD-L1 siRNA (si-PD-L1) using Lipofectamine 2000 (Life Technologies). The sequence for PD-L1 siRNA was as follows:5′-CCAGCACACUGAGAAUCAATT-3′). After 24 h, the cells were treated with or without 10 ng/ml TGF-β1. Finally, the luciferase activity of the transfected cells was determined using a Dual-Luciferase Reporter Assay kit (Promega). Each experiment was performed in triplicate.

### In vivo metastasis model

Sixteen mice were divided equally into two groups termed the PD-L1 silencing group and the control group (8 mice per group). Stable HCC827 cells with the knockdown of PD-L1 (sh-PD-L1–1) or stable negative control cells (Sh-NC) were intravenously (i.v.) injected (1 × 10^6^ cells per mouse) into the tail veins of the mice in the two groups. Mouse TGF-β1 (4 mg/kg bodyweight) was intraperitoneally (i.p.) injected every 5 days after the cell inoculation. The mice were sacrificed after 50 days of inoculation, and their lung tissues were obtained and fixated in Bolin’s fluid. The number of observable metastatic nodules on the surface of each lung was counted manually. Then, the tissues were histologically analysed with H&E staining for the presence of micrometastases. All animal experiments were carried out in accordance with the Guide for the Care and Use of Experimental Animals of the Experimental Animal Center of Soochow University.

### Statistical analysis

All numerical data are presented as the mean ± standard error of the mean (SEM). Statistical analysis was performed by a two-tailed Student’s t-test, and differences were considered significant at a *P*-value of < 0.05. All statistical analyses were performed using GraphPad Prism 5.02 (GraphPad, San Diego, CA, USA) and SPSS 16.0 software (SPSS, Chicago, IL, USA).

## Results

### Correlation between PD-L1 expression and sensitivity to EGFR-TKIs in EGFR-mutant NSCLC cells

**Fig. 1:**
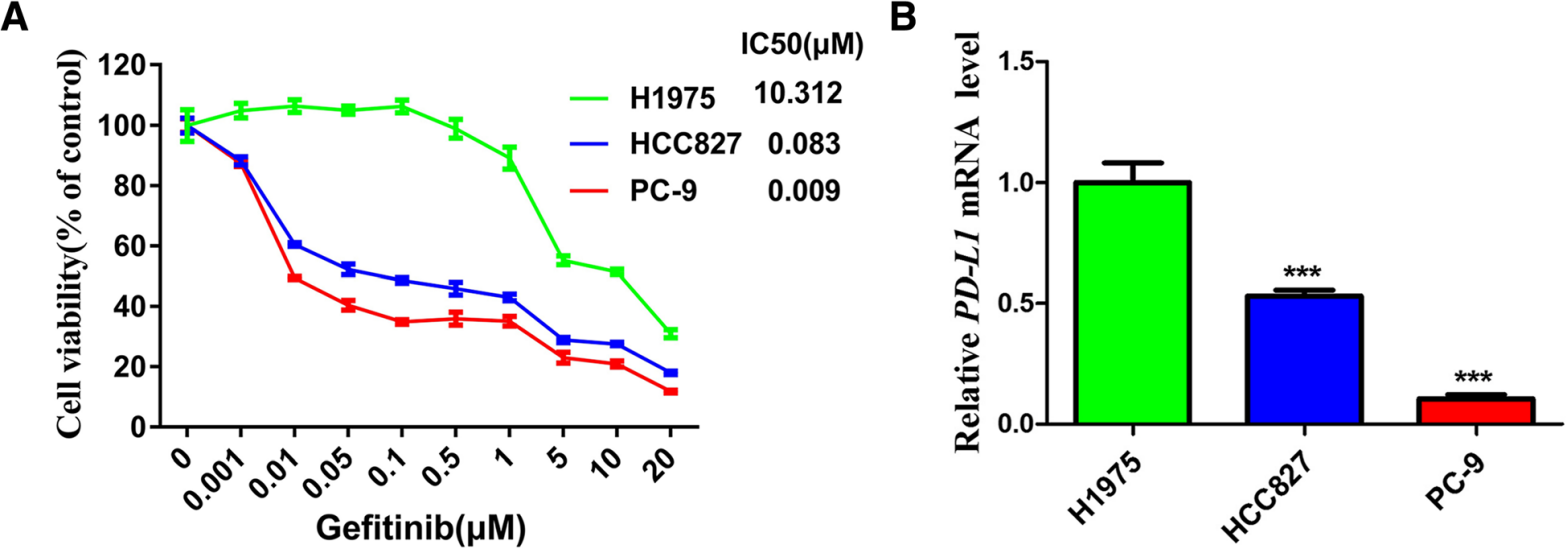
Correlation between PD-L1 expression and sensitivity to EGFR-TKIs in EGFR-mutant NSCLC cells (**a**) Three types of EGFR-mutant NSCLC cells were treated with different concentrations of gefitinib, and the IC50 value was calculated from the dose-response survival curve determined by the CCK-8 assay. **b** The mRNA expression level of PD-L1 in three types of EGFR-mutant NSCLC cells was detected by qRT-PCR. ***P < 0.001

Our previous study demonstrated an EGFR tyrosine kinase domain leucine-to-arginine substitution at codon 858 (p.L858R) and a threonine-to-methionine substitution at codon 790 (p.T790 M) in the H1975 cell line. In addition, an activating deletion in exon 19 was found in the HCC827 and PC-9 cell lines [32]. Three EGFR-mutant NSCLC cell lines were collected to examine the possible association of PD-L1 expression with sensitivity to gefitinib. The CCK-8 assay indicated that the concentration of gefitinib required to inhibit cell viability by 50% (IC50) was highest in H1975 cells (10.312 μM) and lowest in PC-9 cells (0.009 μM) (Fig. 1a). Interestingly, the expression of PD-L1 mRNA in these three cell lines was consistent with the IC50 values for gefitinib: PD-L1 mRNA expression was highest in H1975 cells and lowest in PC-9 cells among the three cell lines (Fig. 1b). These results suggested a link between PD-L1 expression and sensitivity to EGFR-TKIs in EGFR-mutant NSCLC cells.

### PD-L1 is responsible for generating primary resistance to gefitinib

**Fig. 2:**
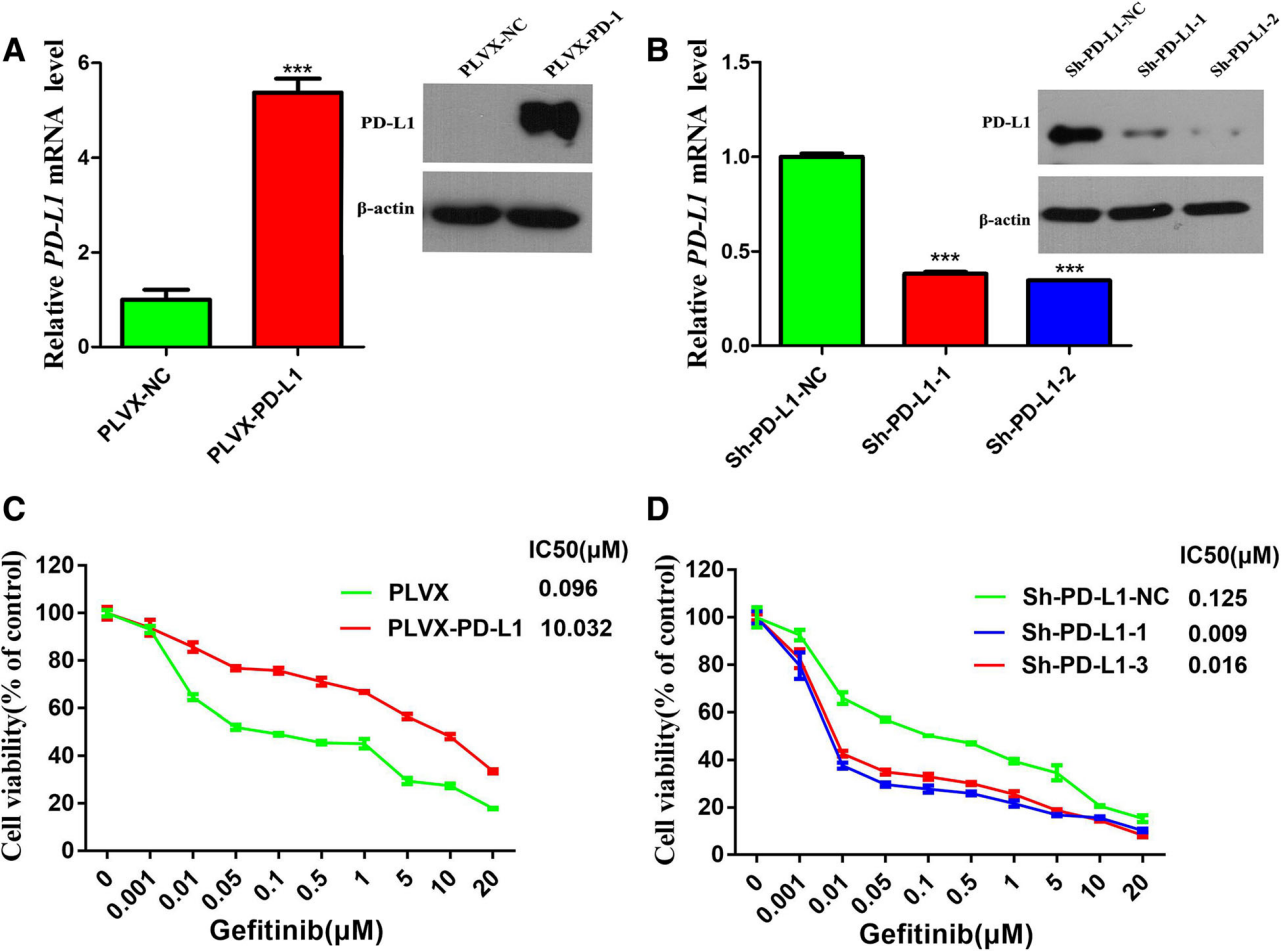
PD-L1 is responsible for generating primary resistance to gefitinib. **a** PD-L1 mRNA and protein expression levels in stable HCC827 cell lines transfected with a PD-L1-expressing plasmid (PLVX-PD-L1) or a negative control (PLVX-NC). **b** PD-L1 mRNA and protein expression levels in stable HCC827 cell lines transfected with two PD-L1 ShRNAs (Sh-PD-L1–1 and Sh-PD-L1–2) or a negative control (Sh-PD-L1-NC). **c** and (**d**) Stable PD-L1-overexpressing and PD-L1-silenced HCC827 cells were treated with different concentrations of gefitinib, and the IC50 value was calculated from the dose-response survival curve determined by the CCK-8 assay. ***P < 0.001

To determine whether PD-L1 mediated primary resistance to EGFR-TKIs in EGFR-mutant NSCLC cells, we overexpressed and knocked down PD-L1 in the HCC827 cell line. The expression of PD-L1 was confirmed by both qRT-PCR and western blotting. The mRNA and protein expression levels of PD-L1 were increased in stable PD-L1-overexpressing HCC827 cells (PLVX-PD-L1) compared to those in HCC827 cells transfected with the plasmid vector (PLVX-NC) (Fig. 2a). The mRNA and protein expression levels of PD-L1 were reduced in stable PD-L1-silenced HCC827 cells (Sh-PD-L1–1 and Sh-PD-L1–2) compared to those in HCC827 cells transfected with a scrambled sequence (Sh-PD-L1-NC) (Fig. 2b). We next assessed the effect of PD-L1 overexpression and knockdown on resistance to EGFR-TKIs in EGFR-mutant NSCLC cells. As shown in Fig. 2c and d, the IC50 value for gefitinib was increased in PD-L1-overexpressing HCC827 cells compared to that in the negative control cells, and the IC50 values for gefitinib were reduced in PD-L1-silenced HCC827 cells compared to those in the negative control cells. These results indicated that PD-L1 is responsible for generating gefitinib resistance in EGFR-mutant NSCLC cells.

### Overexpression of PD-L1 attenuated sensitivity to gefitinib in vivo

**Fig. 3:**
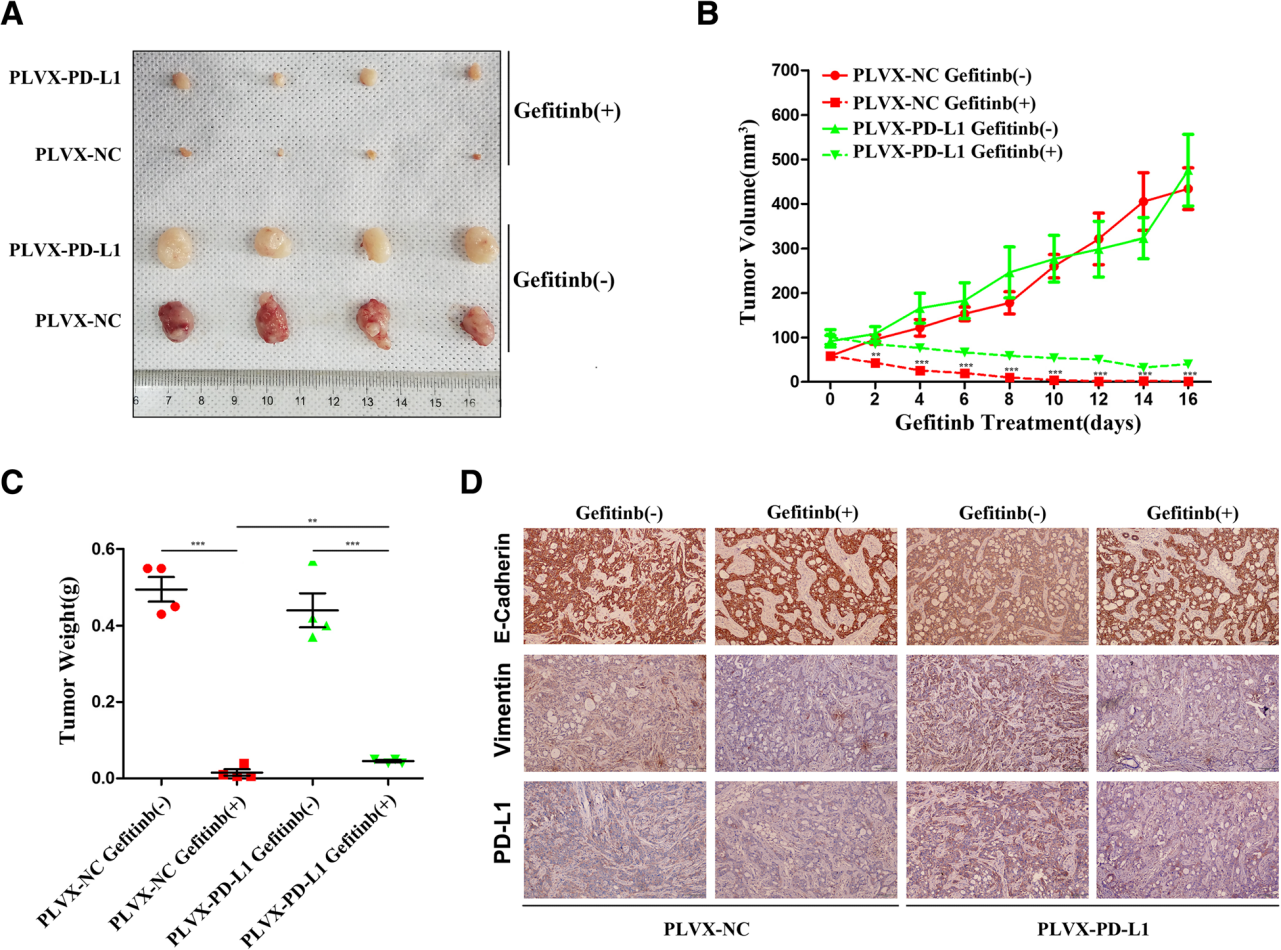
Overexpression of PD-L1 attenuated sensitivity to gefitinib in vivo*.* **a** The tumour volume was measured at the indicated time intervals and calculated. See the Methods for details. **b** and (**c**) At the end of treatment, the tumours were excised, photographed as indicated and weighed. **d** Immunohistochemical staining of PD-L1, E-cadherin and vimentin was quantified based on staining intensity. *P < 0.05; **P < 0.01; ***P < 0.001

To further assess the ability of PD-L1 to mediate primary resistance to gefitinib, we tested the effect of PD-L1 overexpression on sensitivity to gefitinib in a mouse xenograft model. Stable PD-L1-overexpressing HCC827 cells (PLVX-PD-L1) or negative control cells (PLVX-NC) were inoculated subcutaneously into BALB/C athymic mice. Gefitinib (50 mg/kg) or vehicle was administered by oral gavage every 2 days for 16 days. The tumour volumes in the negative control group were larger than those in the PD-L1 overexpression group without gefitinib treatment, but this difference was not statistically significant (Fig. 3a and b). Compared to those in the negative control group, the tumours in the PD-L1-overexpressing group were significantly less sensitive to gefitinib, with larger tumour volumes and weights after gefitinib treatment for 16 days (Fig. 3a-c). Moreover, the resected tissues from the treated xenograft tumours were analysed by IHC to confirm the expression of PD-L1, E-cadherin and vimentin (Fig. 3d). These results suggested that the overexpression of PD-L1 attenuated sensitivity to gefitinib in vivo.

### PD-L1 induced EMT and promoted migration and invasion

**Fig. 4:**
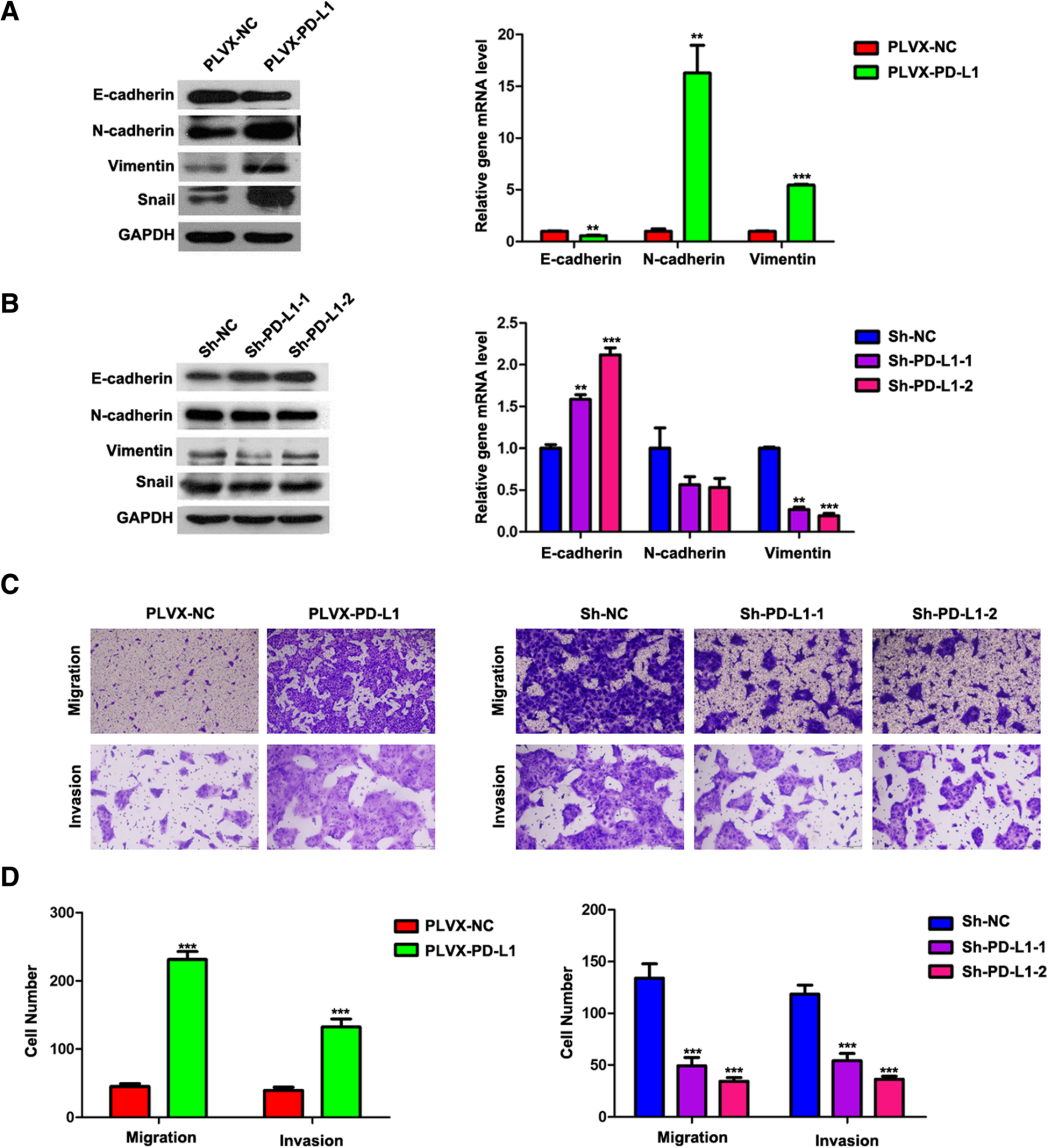
PD-L1 induced EMT and promoted migration and invasion. **a** and (**b**) E-cadherin, N- cadherin, vimentin and Snail mRNA and protein expression levels in stable PD-L1-overexpressing or PD-L1-silenced HCC827 cells were detected by qRT-PCR and western blotting, respectively. **c** and (**d**) Stable PD-L1-overexpressing or PD-L1-silenced HCC827 cells were allowed to migrate through an 8 μM pore Transwell insert, and the migrated cells were stained and counted in at least three microscopic fields (magnification, 100×). The cells were allowed to invade through Matrigel-coated Transwell membranes, and invasive cells were stained and counted under a light microscope. *P < 0.05; **P < 0.01; ***P < 0.001

EMT decreases the clinical activity of gefitinib and erlotinib and the reduces the sensitivity to these compounds in NSCLC cells [25, 26]. To investigate whether PD-L1 may be responsible for primary resistance to EGFR-TKIs through inducing EMT, we analysed the relationship between PD-L1 expression and various EMT markers, including E-cadherin, N-cadherin, vimentin and Snail. The protein expression levels of N-cadherin, vimentin and Snail were increased, while the protein expression levels of E-cadherin were decreased in PD-L1-overexpressing HCC827 cells (PLVX-PD-L1) compared to those in PLVX-NC cells (Fig. 4a). The protein expression levels of N-cadherin, vimentin and Snail were decreased, while the protein expression levels of E-cadherin were increased in PD-L1-silenced HCC827 cells (Sh-PD-L1–1 and Sh-PD-L1–2) (Fig. 4b) compared to those in Sh-NC cells. Similar results were obtained at the mRNA level, except for Snail (Fig. 4a and b). To further confirm that PD-L1 could induce EMT, Transwell assays were performed to detect the effect of PD-L1 on the migratory and invasive abilities in HCC827 cells. PD-L1 overexpression promoted the migration and invasion of HCC827 cells (Fig. 4c); in contrast, PD-L1 knockdown inhibited the migration and invasion of HCC827 cells (Fig. 4d). These results suggest that PD-L1 may be responsible for primary resistance to EGFR-TKIs through the induction of EMT.

### PD-L1 induced EMT by upregulating Smad3 phosphorylation

**Fig. 5:**
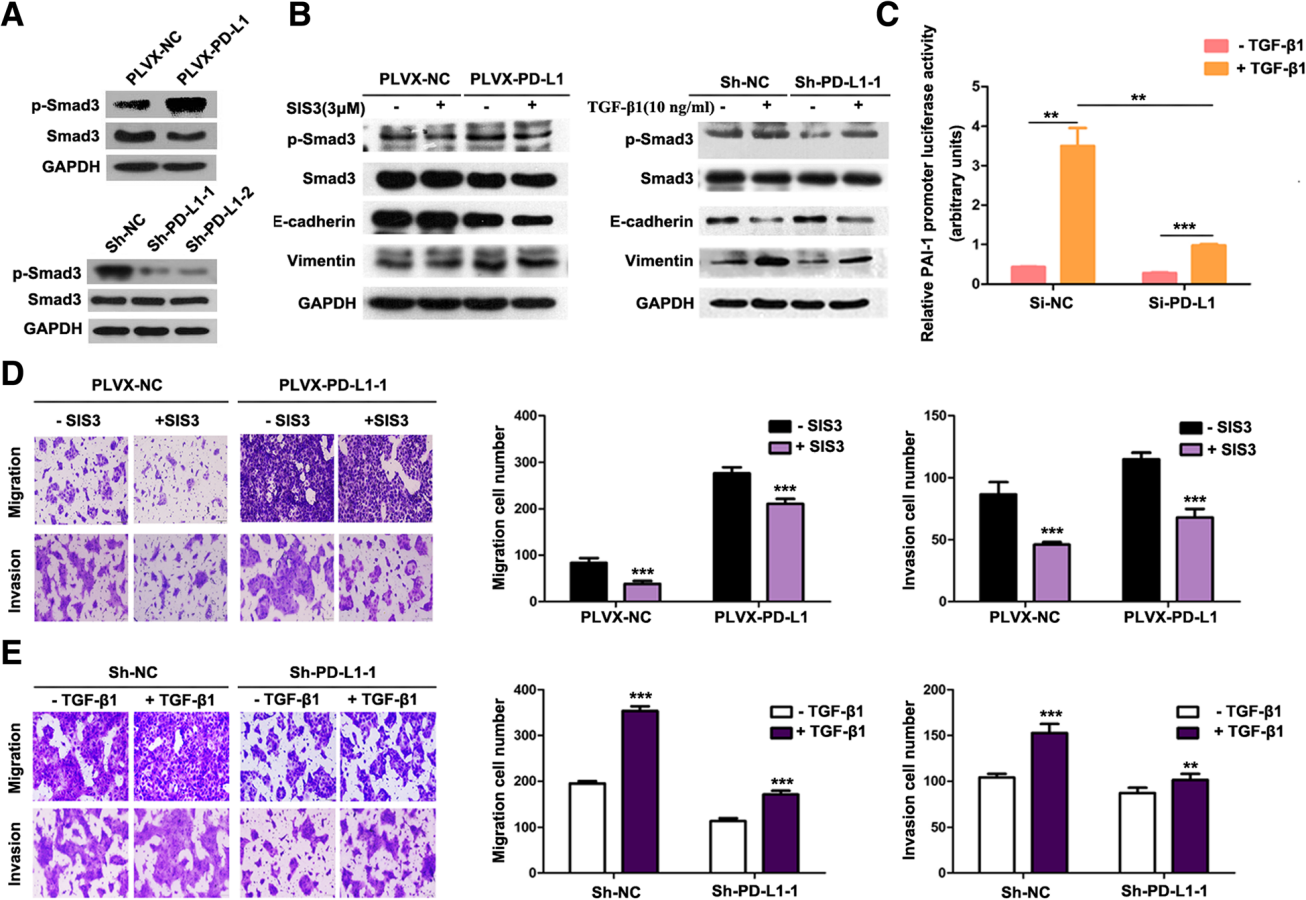
PD-L1 induced EMT by upregulating Smad3 phosphorylation. **a** Western blot analysis of p-Smad3 and Smad3 in stable PD-L1-overexpressing or PD-L1-silenced HCC827 cells. The PD-L1 blot is the same blot shown in Fig. 2a and b. **b** After serum starvation for 24 h, PD-L1-overexpressing and PD-L1-silenced HCC827 cells were treated with SIS3(3 μM) or TGF-β1 (10 ng/ml) for 24 h. The expression of p-Smad3, Smad3, E-cadherin and vimentin was analysed by western blotting. **c** Knockdown of PD-L1 moderated TGF-β1-induced PAI-1 promoter activation. The relative PAI-1 promoter activities of PD-L1-silenced HCC827 cell lines were measured by a Dual-Luciferase Reporter Assay kit. Relative luciferase activity was expressed as the mean fold change from the basal level ± SD of three independent experiments. **d** and (**e**) Stable PD-L1-overexpressing and PD-L1-silenced HCC827 cells were treated with or without SIS3(3 μM) and TGF-β (10 ng/ml) for 24 h, respectively, and allowed to migrate through 8-μM pore Transwell inserts. The migrated cells were stained and counted in at least three microscopic fields (magnification, 100×). The cells were allowed to invade through Matrigel-coated Transwell membranes, and invasive cells were stained and counted under a light microscope

Given that the transforming growth factor (TGF)-β/Smad signalling pathway plays an important role in EMT progression in various epithelial cell types [27, 28], and Smad3 is a key regulator of the canonical TGF-β signalling pathway [29], we hypothesized that Smad3 may play an important role in PD-L1-mediated primary resistance to EGFR-TKIs. To determine whether PD-L1 expression was associated with Smad3, the expression of p-Smad3 and Smad3 in stable PD-L1-overexpressing and PD-L1-silenced HCC827 cells was detected by western blotting. As expected, the increased phosphorylation of Smad3 was observed in PD-L1-overexpressing HCC827 cells compared to that in the negative control cells; in addition, PD-L1 knockdown decreased the phosphorylation of Smad3 in HCC827 cells (Fig. 5a). As shown in Fig. 5b, upon SIS3 (Smad3 inhibitor) treatment, the overexpression of PD-L1 weakened the SIS3-induced downregulation of Smad3 phosphorylation and the upregulation of E-cadherin. Upon TGF-β1 stimulation, the knockdown of PD-L1 inhibited the TGF-β1-induced downregulation of E-cadherin and the upregulation of Smad3 phosphorylation and vimentin. Since PAI-1 is a major downstream target of the TGF-β1/Smad signalling pathway and activated Smad3 can regulate the expression of PAI-1 by combining the cis-acting elements of the PAI-1 promoter region [33, 34], we co-transfected HCC827 cells with PAI-1 reporter plasmid and PD-L1 siRNA to confirm the effect of PD-L1 on the TGF-β/Smad signalling pathway. As shown in Fig. 5c, TGF-β1-induced PAI-1 promoter activation was significantly moderated by PD-L1 knockdown. Furthermore, Transwell assays indicated that PD-L1 overexpression weakened the ability of SIS3 to reduce the migratory and invasive abilities of HCC827 cells (Fig. 5d). In addition, PD-L1 knockdown inhibited the TGF-β1-induced migration and invasion of HCC827 cells (Fig. 5e). These results suggested that PD-L1 induced EMT by activating the TGF-β/Smad signalling pathway.

### Knockdown of PD-L1 inhibited cell metastasis in vivo

**Fig. 6:**
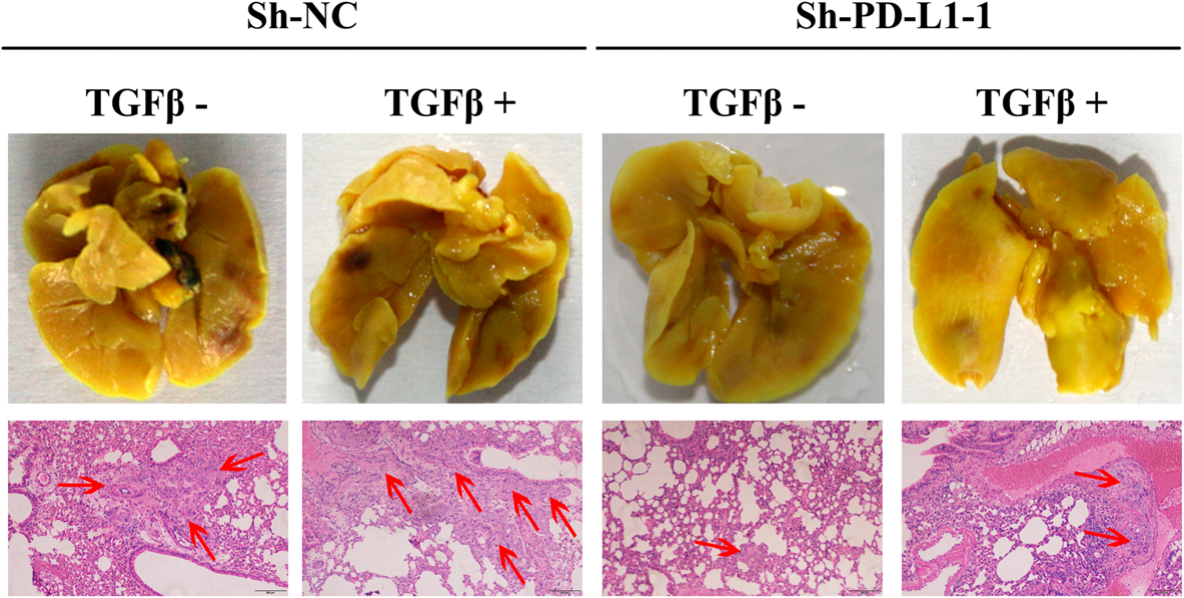
Knockdown of PD-L1 inhibits cell migration and invasion in vivo. Surgically resected lung tissues were fixed in Bouin’s fluid, and representative images of micrometastases detected by H&E staining are presented; red arrowheads indicate micrometastases

To evaluate the effect of PD-L1 knockdown on the migratory and invasive abilities of NSCLC cells in vivo, stable PD-L1 knockdown HCC827 cells (Sh-PD-L1–1) or negative control cells (Sh-NC) were injected into the tail veins of BALB/c athymic nude mice. Mouse TGF-β1 was intraperitoneally injected following cell inoculation. At the end of the experiment, we surgically resected the lung tissues and then fixed and stained the tissues with Bouin’s fluid. As shown in Fig. 6, there were no obvious visible pulmonary metastatic nodules on the surface of the lung tissue, but we detected pulmonary micrometastases by haematoxylin-eosin (H&E) staining. The results showed that fewer and smaller pulmonary micrometastases were detected in the mice injected with stable PD-L1 knockdown HCC827 cells than in the negative control mice. Additionally, compared to the negative control, the knockdown of PD-L1 inhibited TGF-β1-induced HCC827 cell metastasis. These results confirmed that PD-L1 knockdown inhibited the migratory and invasive abilities of HCC827 cells.

## Discussion

EGFR-TKIs have contributed significantly to the successful treatment of NSCLC patients and are approved as a front-line treatment for NSCLC patients harbouring EGFR mutations [35]. However, approximately 30% of patients with EGFR-activating mutations do not respond to EGFR-TKIs (primary resistance) [5, 6]. Although the potential mechanisms of primary resistance, including a coexisting de novo T790 M mutation [9], de novo MET amplification [10], phosphatase and tensin homologue (PTEN) loss [11] and Kirsten rat sarcoma viral oncogene homologue (KRAS) mutations [12], have been investigated in some retrospective analyses and preclinical studies, little is known about the molecular mechanisms involved in primary resistance to EGFR-TKIs. In this study, we found that PD-L1 expression correlates with sensitivity to gefitinib in EGFR-mutant NSCLC cells. Moreover, PD-L1 led to primary resistance to gefitinib by inducing EMT via the activation of the TGF-β/Smad signalling pathway. To the best of our knowledge, this study is the first to investigate the mechanism by which PD-L1 induces primary resistance to EGFR-TKIs in EGFR-mutant NSCLC cells. These results suggest that NSCLC patients with primary resistance to EGFR-TKIs may benefit from PD-L1-targeting immunotherapy.

The present study revealed that the overexpression of PD-L1 reduces sensitivity to gefitinib in EGFR -mutant NSCLC cells. Several studies have investigated the relationship between PD-L1 expression on tumour cells and outcomes in EGFR-mutant patients treated with EGFR-TKIs [36–40], although the conclusions were contradictory. Su et al. [36] showed that strong PD-L1 expression predicted poor response and primary resistance to EGFR-TKIs among 101 EGFR-mutant NSCLC patients naïve to EGFR-TKI treatment. These results were consistent with the results of our study. In contrast, previous studies have reported no significant correlation between PD-L1 expression and the objective response rate (ORR) of EGFR-TKIs [37–39]. There are several potential explanations for the differences between these findings. First, the sample sizes of the investigations varied; larger sample sizes may enhance analytical power and credibility. Second, previous treatment could affect PD-L1 expression. Finally, the characteristics of the enrolled patients may have an impact on the final results. Considering these contradictory findings, reasonable and standardized methods should be used for further verification.

Several studies have reported that PD-L1 performs multiple intracellular functions in various cancer cells. For instance, PD-L1 regulates tumour glucose metabolism [15], prevents cell proliferation and apoptosis [17] and reduces chemotherapy-mediated tumour killing by modifying mitogen-activated protein kinase signals [16]. Similarly, we found that PD-L1 enhanced EMT and promoted migration and invasion by upregulating Smad3 phosphorylation in HCC827 cells. Kim et al. [41] reported that PD-L1 positivity was significantly higher in patients with mesenchymal and epithelial-mesenchymal phenotypes. Moreover, other investigators have also reported associations between PD-L1 expression and EMT in head and neck squamous cancer cells [42], oesophageal cancer [31] and multiforme malignancy [43]. These observations suggested that PD-L1 plays an important role in EMT. EMT is a process by which epithelial cells lose polarity and trans-differentiate into a mesenchymal phenotype; this process has been considered to reduce the clinical activity of gefitinib and erlotinib and the sensitivity of NSCLC cells to these drugs [25, 26].These results suggested that PD-L1-targeting immunotherapy in NSCLC patients with primary resistance to EGFR-TKIs might restore sensitivity to EGFR-TKIs by reversing the EMT phenotype.

In conclusion, this study revealed that positive PD-L1 expression might predict the emergence of primary resistance to EGFR-TKIs. The mechanism by which PD-L1 confers primary resistance to EGFR-TKIs may involve the induction of EMT via the activation of the TGF-β/Smad signalling pathway.

## Data Availability

The authors will make readily reproducible materials described in the manuscript, including software, databases and all relevant raw data are freely available to scientists.

## Abbreviations

CCK-8: Cell counting kit-8
EMT: Epithelial-to-mesenchymal transition
KRAS: Kirsten rat sarcoma viral oncogene
MET: Mesenchymal-epithelial transition
NSCLC: Non-small cell lung cancer
ORR: Objective response rate
PTEN: Phosphatase and tensin homologue
TGF: Transforming growth factor
TKIs: Tyrosine kinase inhibitors
